# *Coccidioides posadasii* in a Dog With Cervical Dissemination Complicated by Esophageal Fistula

**DOI:** 10.3389/fvets.2020.00285

**Published:** 2020-05-19

**Authors:** Adrien Izquierdo, Jared A. Jaffey, Stephanie Szabo, Jason Struthers, Ogi Okwumabua, Eric T. Hostnik, Mana Ohkura, Hien Trinh, Lisa F. Shubitz, Marc J. Orbach, Mary E. White

**Affiliations:** ^1^Department of Specialty Medicine, College of Veterinary Medicine, Midwestern University, Glendale, AZ, United States; ^2^Department of Pathology and Population Medicine, College of Veterinary Medicine, Midwestern University, Glendale, AZ, United States; ^3^Department of Veterinary Clinical Sciences, College of Veterinary Medicine, The Ohio State University, Columbus, OH, United States; ^4^School of Plant Sciences, University of Arizona, Tucson, AZ, United States; ^5^Valley Fever Center for Excellence, University of Arizona College of Medicine-Tucson, Tucson, AZ, United States

**Keywords:** valley fever, canine, coccidioidomycosis, draining tract, esophageal fistula, thyroiditis, coccidioides

## Abstract

A 5-year-old male, neutered mixed breed dog with a history of a mass with an associated draining tract on the ventral cervical region was diagnosed with an esophageal fistula. The dog exhibited serosanguinous discharge from the draining tract, with enlarged left superficial cervical and mandibular lymph nodes, and was reported to have difficulty with deglutition of solid foods. Computed tomography revealed a communication of the draining tract with the esophagus along with enlargement of the left lateral retropharyngeal, left medial retropharyngeal, and mandibular lymph nodes. This prompted surgical exploration and debridement of the site, with closure of the esophageal fistula. Histopathology of thyroid gland, skeletal muscle, and adipose tissue obtained during surgical exploration showed spherules consistent with *Coccidioides spp*. infection. Antibody titers performed post-operatively were consistent with an active *Coccidioides* spp. Infection. By fungal culture and subsequent PCR and DNA sequencing, *C. posadasii* was identified as the species infecting the dog. Over the course of 85 days of antifungal therapy, discharge from the draining tract, lymphadenomegaly, and cutaneous and subcutaneous nodules resolved. In conclusion, this is the first reported case of disseminated coccidioidomycosis to the cervical region of a dog with involvement of the thyroid gland, skeletal muscle, adipose tissue, connective tissue, and secondary esophageal fistula. *Coccidioides* spp. infections should be considered a differential diagnosis in unusual cases for dogs that live in or have traveled to endemic areas.

## Background

Coccidioidomycosis is the most common systemic fungal disease in the southwestern United States, and is caused by the dimorphic, saprobic fungi, *C. immitis* or *C. posadasii* (1). The organism is endemic in dry climates of Arizona, California, Texas, New Mexico, Mexico, and Central and South America (2). The incidence of infection in humans within endemic regions has increased over the last 10 years (3, 4). Similarly, an increased incidence of disease is presumed to have also occurred in dogs. A community-based longitudinal and cross-sectional study found that dogs raised in Pima and Maricopa counties in Arizona had a high risk of infection (28%) by 2 years of age (5).

Coccidioidomycosis in dogs can manifest as primary respiratory or disseminated disease (6). This heterogeneous distribution of systemic involvement results in a variety of clinical signs and severity of disease ranging from mild respiratory disease, to vague chronic signs (e.g., fever, lethargy, anorexia, weight loss, limping), and even severe life-threatening illness (7). Severe systemic disease is more likely in cases with disseminated infection, which occurs in ~20% of dogs (8). In dogs, *Coccidioides* can disseminate to the central nervous system, lymph nodes, skin, bone, pericardium, eye, testes, prostate, and other parenchymal organs including the liver, spleen, kidneys, and gastrointestinal tract (6). In humans, rare sites of dissemination include deep subcutaneous tissue in the cervical region, larynx, and thyroid gland (9).

The diverse array of potential regions of dissemination can lead to unusual but important manifestations of disease. One such example in humans is cervical *C. immitis* lymphadenitis resulting in fistula formation to the esophagus (10). We report the first case, to the best of our knowledge, of *Coccidioides* dissemination in a dog to thyroid gland, adipose tissue, skeletal muscle, lymph node, and connective tissue resulting in fistula formation to the esophagus. This case highlights a novel manifestation of *Coccidioides* infection as well as the need to include this fungal infection as a differential diagnosis for unusual cases in dogs that live in or have traveled to endemic regions.

## Case Presentation

A 5-year-old male neutered, Heeler mixed breed dog (23.6 kg) presented to Midwestern University College of Veterinary Medicine Companion Animal Clinic (MWU-CAC) for evaluation of a subcutaneous mass with a draining tract in the ventral cervical region (day 1). The dog had not displayed any coughing, lethargy or weight loss, however some difficulty swallowing solid food was reported by the owner. The dog could reportedly prehend and chew kibble normally, however, appeared to have difficulty with deglutition. He had lived in Arizona for 3 years and frequently hiked with the owners and was allowed unsupervised time in their yard. The owner first identified a small subcutaneous mass in the region of the right ventral neck 8 months before presentation to the MWU-CAC. This mass enlarged over the next month and a draining tract with thick, purulent discharge subsequently developed. Two months later the primary care veterinarian performed a complete blood count, biochemistry profile, total T4, and a fine needle aspirate of the mass. Biochemical abnormalities included hyperglobulinemia (5.1 g/dL; reference interval 2.4–4.0 g/dL) and hypoalbuminemia (2.6 g/dL; reference interval 2.7–3.9 g/dL). The total thyroxine was within the reference interval (1.4 μg/dL; reference interval 1–4 μg/dL) and the complete blood count did not reveal any abnormalities. Fine needle aspiration of the neck mass performed by the primary care veterinarian revealed marked pyogranulomatous inflammation without infectious organisms or neoplastic cells. The primary care veterinarian treated the dog with successive courses of oral clindamycin (4 weeks) and ciprofloxacin (2 weeks). The administration of these antibiotics resulted in transient reductions in mass size and discharge, but never resolved entirely.

Physical examination findings at MWU-CAC included heart rate of 90 beats/minute, respiratory rate of 30 breaths/minute, and a normal temperature 101.1°F (38.4°C). Cardiothoracic auscultation did not reveal a murmur, arrhythmia, or abnormal lung sounds. The left mandibular and superficial cervical lymph nodes were enlarged (2 × 2 cm) and firm. A 3 × 3 cm, firm, subcutaneous mass was identified in the left submandibular region. There was a small focal region along the ventral aspect of the mass that actively drained serosanguinous fluid. Differential diagnoses considered at that time included lymphadenitis, sialadenitis, salivary mucocele, neoplasia, and coccidioidomycosis.

**Figure 1:**
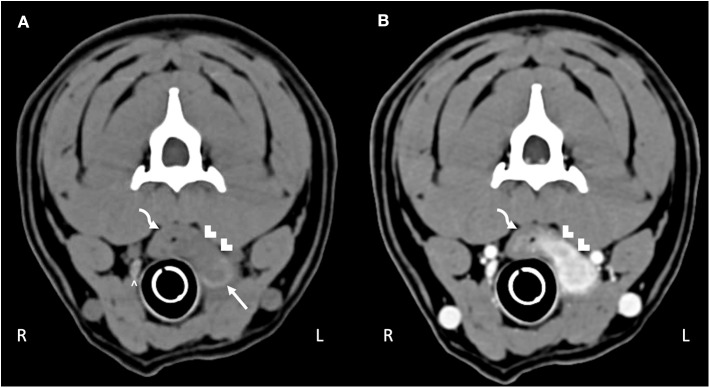
Computed tomography of the **(A)** pre-contrast and **(B)** post-contrast neck. The curved arrow highlights the esophagus. Hypoattenuating, contrast-enhancing tissue obscures the mucosal tissue extending through the esophageal wall into the surrounding tissue (white chevrons). The abnormal tissue effaces the left thyroid lobe (white arrow); compare to the normal right thyroid lobe (white caret).

**Figure 2:**
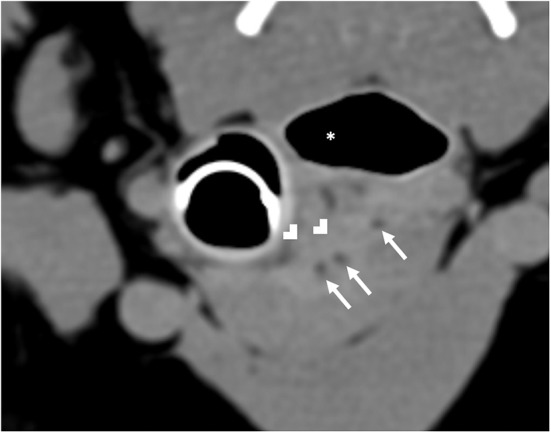
Computed tomography of hyperattenuating soft tissue (white chevrons) representing the fistulous tract that is contiguous with the esophagus (white asterisk). Within the fistulous tract there are small gas bubbles (white arrows).

**Figure 3:**
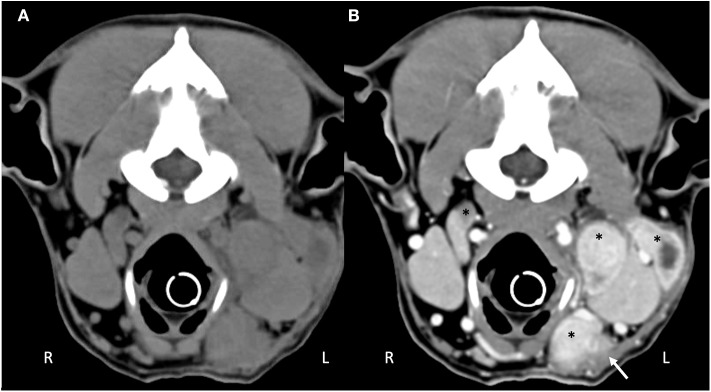
Computed tomography of the **(A)** pre-contrast and **(B)** post-contrast cranial neck. There are multiple enlarged, contrast enhancing lymphocenters that are primarily left-sided (black asterisks). Central portions of the lymphocenters are heterogeneous and contrast-enhancing with hypoattenuating regions. Soft tissue striations surround the enlarged lymphocenters causing disruption of the fat fascial planes between structures (white arrow).

Computed tomography (CT) imaging of the skull and neck was subsequently performed on day 10 in order to better understand the extent of disease and origin of the mass. Helical images of the skull were acquired with a slice thickness of 1.00 mm and a pitch of 0.85 was used. There was abnormal pre-contrast hypoattenuating tissue that was heterogeneously contrast-enhancing associated with the left aspect of the esophagus (Figures 1A,B). The enhancing tissue was confluent with and obscured margins of the contrast-enhancing tissue of the esophageal mucosa. The enhancing tissue extended beyond the border of the esophagus into the soft tissues of the neck, obscuring margins of the left lobe of the thyroid gland. The tissue that extended from the esophagus was hyperattenuating on the pre-contrast series with small gas bubbles within the hyperattenuating tissue (Figure 2). The lymph nodes of the left side of the head and neck were enlarged with heterogeneous contrast enhancement of the central portion of the lymph nodes and strong peripheral contrast enhancement giving a rim-enhancing appearance; affected lymph nodes included left medial retropharyngeal lymph node, left lateral retropharyngeal lymph node, and left mandibular lymph nodes (Figures 3A,B). There was a focal hypoattenuating region of the craniodorsal aspect of the right medial retropharyngeal lymph node. There were linear soft tissue striations that surrounded the affected lymph nodes resulting in disruption of the fat planes between the lymph nodes, left mandibular salivary gland, and the region muscle bellies.

**Figure 4:**
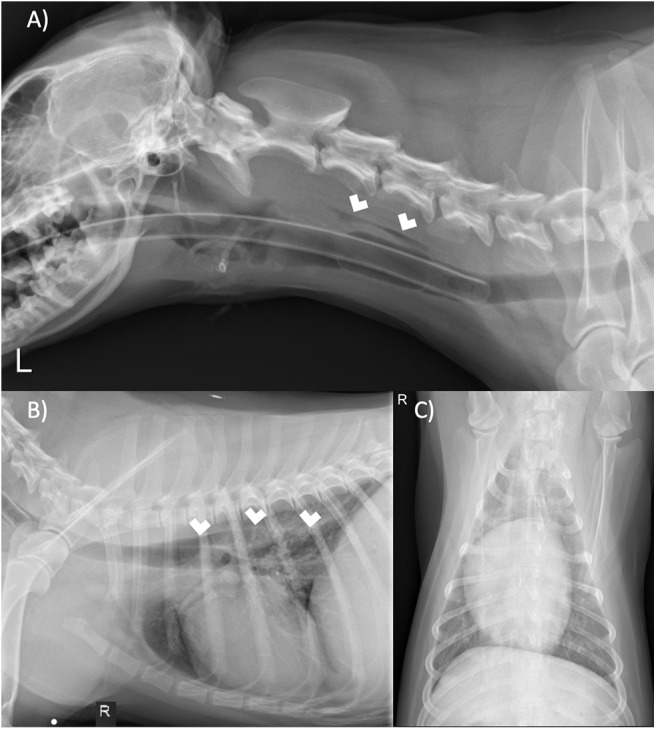
**(A)** Left lateral neck, **(B)** right lateral thorax, and **(C)** ventrodorsal thorax radiographs. There is gas within the cervical and thoracic esophagus (white arrows). The remaining tissues of the neck and thorax are unremarkable.

**Figure 5:**
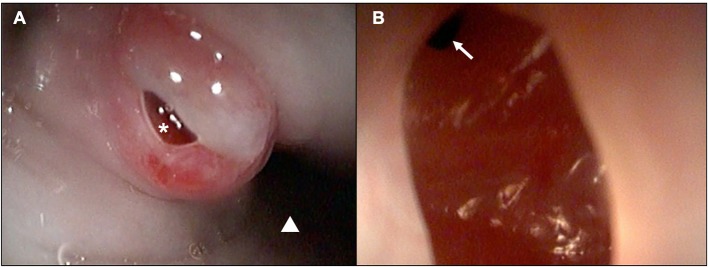
Esophagoscopic images demonstrating a **(A)** chronic-appearing, erythematous fistulous tract (white asterisk) ~3cm distal to the upper esophageal sphincter (white triangle; distal esophagus) and **(B)** visualization within the fistula and distal extension of the tract (white arrow).

These results prompted the recommendation of CT imaging of the thorax, but this was declined by the owner in favor of thoracic/neck radiographs and endoscopic evaluation to visually assess the extent of esophageal involvement. Radiographs revealed a mild amount of gas within the cervical and intrathoracic portions of the esophagus (Figure 4). The cardiovascular structures, pulmonary parenchyma, pleural, and osseous structures were unremarkable. Esophagoscopy revealed a small hole opening in the esophagus surrounded by proliferative tissue (Figure 5).

**Figure 6:**
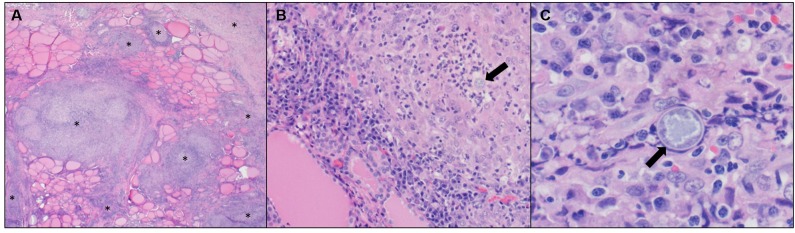
**(A)** Neck mass. Thyroid gland is disrupted and replaced by multifocal to coalescing pyogranulomatous and lymphoplasmacytic inflammation with peripheral fibroplasia (*). Hematoxylin and eosin (H&E), 2X objective. **(B)** Neck mass. Centrally, pyogranulomas occasionally have fungal spherules (arrow). H&E, 20X objective. **(C)** Neck mass. Intralesional fungal spherules are circular, measure up to 21 μm, and have a 1 μm refractile capsule with central vacuolated basophilic granular material (arrow). H&E, 60X objective.

Surgical exploration of the ventral neck was pursued on day 15 due to the chronic draining tract on the left side of the neck at the level of the proximal trachea and the endoscopic finding of a left-sided esophageal defect at the level of the 3rd tracheal ring. A ventral midline cervical incision was made from the larynx to the manubrium. The paratracheal fascia and left sternothyroid muscle were retracted, and the draining tract was traced to a 1 cm tan, fibrous lesion over the left lobe of the left thyroid gland. Blunt and sharp dissection of the fibrosis exposed an adhesion to the esophagus at the level of the 3rd tracheal ring. Upon blunt dissection of the adhesion, a 5 mm full-thickness defect in the esophagus was exposed. The edges of the defect were debrided 1 mm circumferentially and then closed with full-thickness simple interrupted sutures of 3-0 polydioxanone. The remaining tan, fibrous tissue of the draining track was resected and the ventral neck was copiously lavaged with sterile saline. A ¼ inch penrose drain was placed in the left ventral neck exiting just caudal and lateral to the cervical incision. Two incisional biopsies collected from the neck were submitted for histopathology and fixed in 10% neutral buffered formalin. Representative sections were taken, routinely processed and paraffin embedded, and stained with hematoxylin and eosin. Microscopically, thyroid gland and peripheral adipose tissue, skeletal muscle, and connective tissue were infiltrated and disrupted by multifocal to coalescing mixed inflammation often organized as pyogranulomas rimmed by lymphocytes, plasma cells, and surrounded by dense fibroplasia transitioning to peripheral edematous granulation tissue (Figure 6). Centrally, often admixed with cell debris and neutrophils, there were occasional circular up to 21 μm diameter fungal structures with a 1 μm refractile capsule and central vacuolated basophilic granular material (*Coccidioides* spp. spherules) (Figure 6).

Aspirated fluid from the draining tract was plated on glucose-yeast (GYE) agar and incubated at 37°C in ambient air under Biosafety Level 3 containment. Two fungal colonies grew and were subcultured (strains A and B) for DNA extraction as described (11). PCR primers ITS1F and LR3 (12, 13) were used to amplify a 1.2 kb region that included the nrITS region as well as 600 bp of the 28S ribosomal large subunit gene, and the amplicons were sequenced in order to define the species of *Coccidioides* present in the aspirate. The PCRs were performed in 20 μL using Promega GoTaq® Green Master Mix (Madison, WI), following the manufacturer's protocol. Amplicons were sequenced at Eton Biosciences Inc. (San Diego, CA). Sequences were edited and assembled using SeqTrace 0.9.0 (14) and then aligned against ITS sequences of type specimens of *C. immitis* (strain CBS 120936; GenBank accession NR_157446.1) and *C. posadasii* (strain ATCC 28868 = Silveira; GenBank accession NR_145259.1) using MUSCLE (15). The alignment was viewed in Mesquite (16) to analyze phylogenetically informative sites. Both sequences that distinguish *C. immitis* and *C. posadasii* has been previously described (17). Table 1 shows that both isolates have nrITS sequences that match the *C. posadasii* at all of the phylogenetically informative sites, indicating that the infection was caused by *C. posadasii*, consistent with the area of residence of the dog.

**Table 1:** Sequences at phylogenetically informative sites of the nrITS region.

	**ITS1 base positions**			**ITS2 base positions**			
	**37**	**74**	**158^**c**^**	**34**	**37**	**148**	**157–161**
Clinical isolate strain A	T	C	C	C	-	C	ATT-T
Clinical isolate strain B	T	C	C	C	-	C	ATT-T
*Coccidioides immitis* type CBS 120936	-^b^	T	T	T	-	T	T—A
*Coccidioides posadasii* type ATCC 28868	T	C	C	C	-	C	ATT-A
*Coccidioides immitis*^a^	-	T	T	T/C	-	T	T—A/TTWAA/TT-AA/TTTAA
*Coccidioides posadasii*^a^	T	C	C	C	-/A	C	ATT-A/ATT-T

a Sequences at phylogenetically informative sites for C. immitis and C. posadasii as described by Tintelnot et al. (17).

b Dash(-) represents lack of nucleotide at that position.

c *Base position is amended from 125 to 158 in Johnson et al. (31) based on personal communication with K. Tintelnot*.

Serum *Coccidioides* spp. titers were positive at a titer of 1:2 (IgM) and 1:32 (IgG). Medical management at the time of discharge (day 17) included tramadol (3 mg/kg PO Q8–12hr), fluconazole (6 mg/kg PO Q12hr), amoxicillin/clavulanate (15 mg/kg PO Q12hr), and carprofen (2 mg/kg PO Q12hr). The drain was removed 5 days after hospital discharge. At a recheck appointment 2 weeks later (day 37), the surgery site was again draining fluid, which appeared red and opaque. A 3 cm fluctuant, subcutaneous swelling had developed ~4 cm ventral to the previous drain site, where purulent discharge was still observed. The mass dorsal to the drain site had increased from ~2–2.5 cm, and the left mandibular lymph node remained enlarged. The owners reported that the dog had shown good energy levels at home but had a decreased appetite and occasionally coughed. Serum was submitted to Michigan State University Diagnostic Laboratory to assess thyroid function. Despite *Coccidioides* infiltration of left thyroid tissue and subsequent hemithyroidectomy, the dog maintained normal thyroid function; total thyroxine (7 nmol/L; RI 11–60 nmol/l), free T4 by dialysis (16 pmol/L; RI 6–42 pmol/L), thyroid stimulating hormone (0.10 ng/mL; RI 0.00–0.58 ng/mL).

The dog was evaluated again 7 days later (day 44) and new 3 cm by 1.5 cm subcutaneous mass was noted on the craniolateral aspect of right antebrachium. Fine needle aspiration of the mass revealed moderate mixed inflammation and a large, round basophilic structure that appeared to be an empty *Coccidioides* spherule. The left mandibular lymph node and 2 subcutaneous nodules located on the ventral neck along midline remained unchanged in size. The draining tract was scabbed over but was report by the owner to still be draining. Fluid from the draining tract was evaluated cytologically and revealed marked mixed inflammation with occasional extracellular *Coccidioides* spp. spherules. In light of this finding the dosage of fluconazole was increased (7.9 mg/kg PO Q12hr).

On day 85 (the last day of follow-up) the dog was evaluated as part of a routine reassessment. The owners did not report any abnormalities that had transpired since the last examination. Physical examination revealed a new small 1.5 × 1.5 cm nodule on the ventral neck that ruptured and drained a small amount of serosanguious fluid during the examination. Interestingly, the dog had complete resolution of lymphadenomegaly as well as all previously identified nodules and the draining tract on the ventral neck. Fluid from the ruptured nodule was evaluated cytologically and showed marked mixed inflammation with *Coccidioides* spp. spherules. The dosage instructions for fluconazole did not change. Follow-up with the owner by phone (day 145) revealed that the dog had no draining tract wounds and normal deglutition.

## Discussion

To the authors' knowledge, this is the first report describing *Coccidioides* infection in a dog with dissemination to thyroid gland, adipose tissue, skeletal muscle, lymph node, and connective tissue resulting in fistula formation to the esophagus. Esophageal fistulous communication from the neck to the esophagus via an extension of infection (e.g., abscess, granuloma, lymph node) has not been reported in dogs. Acquired esophageal fistulas in dogs are uncommon and tend to occur secondary to esophageal foreign bodies as well as esophageal diverticula (18–21). These esophageal fistulous tracts in dogs most commonly connect with the trachea or bronchi (18–21). Pathogens in people that have been reported to cause fistulous communications with tissues in the neck region include *C. immitis, Candida albicans*, and *Mycobacterium tuberculosis* (10, 22, 23).

Coccidioidomycosis with associated esophageal fistula and lymphadenitis is a rare presentation in people with symptoms including the development of a cervical mass and draining tracts, in addition to more non-specific signs such as coughing, weight loss, fever, and fatigue being reported in these patients (10, 24). The disease otherwise presents similarly in humans as it does in dogs, with pulmonary involvement being the predominant manifestation and disseminated forms being less common (6, 9, 25). The reason coccidioidomycosis in this dog presented as a draining tract and fistulous connection to the esophagus remains unknown, and is likely multifactorial. The clinical signs of *Coccidioides* infection depend on the infectious dose of inhaled arthroconidia and the host immune response (26). Dogs that mount an ineffective immune response early in the course of pulmonary infection are susceptible to dissemination via lymphatics or hematogenously. The dog in this report likely had dissemination to cervical lymph nodes, which then infiltrated local regional tissues. An alternative, albeit unlikely explanation, is that the dog had a direct inoculation of *Coccidioides* in the cervical neck. Direct inoculation occurs rarely, with only one report in the veterinary literature suggestive of this route of infection following the removal of a foxtail from a wound in a dog (27).

In human medicine, cases of cervical coccidioidomycosis have been reported to present with abscesses, granulomatous plaques, laryngeal involvement, masses within the cervical region, mucosal lesions, osteomyelitis of the bones of the skull, and abscess deep within the tissue of the neck (28). Cases of cervical lymphadenopathy can result in enlarged, painful, possibly necrotic submandibular lymph nodes, draining wounds, poor appetite, fatigue, dysphagia, and weight loss (10, 24). Treatment of coccidioidomycosis affecting the cervical region generally consists of antifungal therapy.

Azole antifungal drugs such as fluconazole (5–10 mg/kg BID) and itraconazole (5–10 mg/kg/day) are most commonly used, though amphotericin B is used in cases that are unable to tolerate azole antifungals and that have severe or rapidly progressive disease (6, 8). Debridement of an affected site may be pursued if there is necrotic tissue or a granuloma that must be removed (24, 28, 29). One case highlighting this approach comes from a geriatric human patient with *C. immitis* related esophageal fistula that was treated with surgical resection and fluconazole (10). The dog in this report was noted to have enlarged mandibular and superficial cervical lymph nodes, a subcutaneous mass with an associated draining tract, as well as difficulty eating. It is possible that a diagnosis of coccidioidomycosis could have been made with cytologic evaluation of fluid from the draining tracts or serum *Coccidioides* spp. titers on day 1. However, this diagnosis would not have precluded the pursuit of a CT with subsequent esophagoscopy to better understand the connection between the cervical wounds and dysphagia. Further, at the time surgical correction was pursued, a diagnosis of coccidioidomycosis was unknown; however, would not have changed the decision to pursue surgical correction as the esophageal fistula would still have needed to be closed. While no studies assessing medical management of esophageal fistulas in dogs have been published to the authors knowledge, publications in human medicine indicate that medical management is unlikely to lead to resolution of the fistula (30).

## Conclusion

In conclusion, this report documents the first case of coccidioidomycosis associated with an esophageal fistula in a dog. This case highlights the importance of including coccidioidomycosis as a differential diagnosis for dogs with pyogranulomatous, chronic inflammation of unknown etiology that have lived in or traveled to endemic regions.

## Data Availability Statement

All datasets generated for this study are included in the article/supplementary material.

## Author Contributions

AI, JJ, SS, JS, OO, EH, MO, HT, LS, MJO, and MW: medical diagnosis, writing and editing manuscript, and review of final submission.

## Conflict of Interest

The authors declare that the research was conducted in the absence of any commercial or financial relationships that could be construed as a potential conflict of interest.
